# Progress in Genito-Urinary Surgery

**Published:** 1904-04-02

**Authors:** 


### PROGRESS IN GENITO-URINARY SURGERY.

**The Functional Diagnosis of Kidney Disease.**—F. Engelmann [1] has been able to prove, as Bickel had previously done, that the electrical conductivity of the urine and blood-serum of healthy and unhealthy individuals is extraordinarily constant. Further, the electrical conductivity of the urine obtained from each kidney by ureteral catheterisation is exactly proportionate to, and varies proportionately with the molecular concentration. For example, if one kidney is diseased, the conductivity is diminished just as the freezing-point is raised (*i.e.* brought nearer to that of distilled water). Hence, the determination of the electrical conductivity of urine gives excellent control over the results obtained by cryoscopy.

**Movable Kidney.**—Augustus H. Goelet (New York) [2] describes a technique for fixation of the prolapsed kidney which he has employed in 171 consecutive nephropexies on 134 patients, in 37 of the cases for both kidneys at the same time. In this series of cases there was no death and no failure to secure sufficient fixation of the kidneys. The following points are considered by the author to be essential for success in nephropexy by any method, namely:— (1) Careful preparation of the patient beforehand to prevent retching after the operation. (2) Particular care in completely detaching the colon from the kidney, to prevent subsequent dragging upon it when the bowel becomes distended. (3) Prevention of re-attachment of the colon to the kidney which may be secured by packing a gauze drain below and in front of the kidney, and by evacuating the bowels early. (4) The sustaining sutures must be inserted under the fibrous capsule in such a manner as to prevent them tearing out or cutting through when tied, and the suture material must not stretch or absorb before the kidney becomes fixed. (5) The kidney must be restored by the operation to its normal position, not to a lower position than normal. (6) The fatty capsule must be prevented from coming between the kidney and the structures to which it is sutured. This the author prevents by packing gauze around the lower pole of the kidney. The special features of the author's technique are the following: The patient is placed prone, with an air-cushion under the abdomen. The latissimus dorsi, the layer of fascia beneath it and the transversalis fascia, are separated by blunt hooks, not by the knife. By this means the ilio-hypogastric nerve and the vessels with it are saved. The fibrous capsule of the kidney is not detached, the author considering this quite unnecessary to secure firm adhesions. The sustaining sutures for the kidney are two in number. They are inserted on the posterior aspect of the free border of the kidney, one at about the centre, the other about the junction of the middle and lower thirds. These sutures penetrate only just beneath the fibrous capsule, and are passed first in a downward and inward direction for half an inch and brought out again; then a second time passed, transversely; and lastly passed upwards and brought out at a point about one inch from the first point of entry and opposite to that point. The other suture is similarly passed just above the first. The sutures are said to resist a considerable strain without tearing out. The free ends of the sustaining sutures are carried through all the structures of the back and brought out on the surface at the upper angle of the incision, and tied over folded gauze placed lengthwise to the incision at its upper end. They are not removed before the twentieth day. R. E. Weigall,[3] in the *Australasian Medical Gazette* for November 1903, records a case of gangrene of the kidney due to the twisting of the pedicle, the kidney previously having been loose. The patient, a married woman of 23 years of age, was seized with sickening pain in the right kidney region, followed in an hour by hæmaturia. The right kidney was very tender, and any movement caused pain. After 28 hours there was a severe rigor and the temperature rose to 104.6°. On the fifth day 23 ounces of urine only were passed. The kidney could then be grasped without pain. The temperature kept up and the pulse rate was from 140 to 160 per minute. Gangrenous kidney was diagnosed and an operation performed, though the patient appeared to be moribund. The organ was found to be gangrenous, large, and loose, and completely twisted on its axis. It was removed and the patient recovered.

[1] (Abst.) Zentralb. f. Chur. No. 51. Dec. 19, 1903, p. 1397. [2] Ann. of Surg., Dec. 1903, p. 769. [3] (Abst.) Lancet, Jan. 9, 1904, p. 111.

*(To be continued.)*

